# De Novo Design and In Vitro Testing of Antimicrobial Peptides against Gram-Negative Bacteria

**DOI:** 10.3390/ph12020082

**Published:** 2019-06-03

**Authors:** Boris Vishnepolsky, George Zaalishvili, Margarita Karapetian, Tornike Nasrashvili, Nato Kuljanishvili, Andrei Gabrielian, Alex Rosenthal, Darrell E. Hurt, Michael Tartakovsky, Maya Grigolava, Malak Pirtskhalava

**Affiliations:** 1 Ivane Beritashvili Center of Experimental Biomedicine, 0160 Tbilisi, Georgia; maia.grigolava@science.org.ge; 2 Labarotory of Animal Molecular Biology, Agricultural University of Georgia, 240 David Aghmashenebeli Alley, 0159 Tbilisi, Georgia; gi.zaalishvili@agruni.edu.ge (G.Z.); m.karapetian@agruni.edu.ge (M.K.); tnasr2015@agruni.edu.ge (T.N.); nkulj2014@agruni.edu.ge (N.K.); 3 Office of Cyber Infrastructure and Computational Biology, National Institute of Allergy and Infectious Diseases, National Institutes of Health, Bethesda, MD 20892, USA; gabr@niaid.nih.gov (A.G.); alexr@niaid.nih.gov (A.R.); darrellh@niaid.nih.gov (D.E.H.); mtartakovs@niaid.nih.gov (M.T.)

**Keywords:** antimicrobial peptides, predictive models, drug design

## Abstract

Antimicrobial peptides (AMPs) have been identified as a potentially new class of antibiotics to combat bacterial resistance to conventional drugs. The design of de novo AMPs with high therapeutic indexes, low cost of synthesis, high resistance to proteases and high bioavailability remains a challenge. Such design requires computational modeling of antimicrobial properties. Currently, most computational methods cannot accurately calculate antimicrobial potency against particular strains of bacterial pathogens. We developed a tool for AMP prediction (Special Prediction (SP) tool) and made it available on our Web site (https://dbaasp.org/prediction). Based on this tool, a simple algorithm for the design of de novo AMPs (DSP) was created. We used DSP to design short peptides with high therapeutic indexes against gram-negative bacteria. The predicted peptides have been synthesized and tested in vitro against a panel of gram-negative bacteria, including drug resistant ones. Predicted activity against *Escherichia coli ATCC 25922* was experimentally confirmed for 14 out of 15 peptides. Further improvements for designed peptides included the synthesis of D-enantiomers, which are traditionally used to increase resistance against proteases. One synthetic D-peptide (SP15D) possesses one of the lowest values of minimum inhibitory concentration (MIC) among all DBAASP database short peptides at the time of the submission of this article, while being highly stable against proteases and having a high therapeutic index. The mode of anti-bacterial action, assessed by fluorescence microscopy, shows that SP15D acts similarly to cell penetrating peptides. SP15D can be considered a promising candidate for the development of peptide antibiotics. We plan further exploratory studies with the SP tool, aiming at finding peptides which are active against other pathogenic organisms.

## 1. Introduction

The solution to the problem of bacterial resistance to antibiotics is one of the most pressing tasks in microbiology. Bacterial infections caused by multidrug-resistant (MDR) strains represent a new threat to public health around the world. Gram-negative bacteria cause dangerous diseases such as pneumonia, meningitis, and many others. Lipopolysaccharides, which are highly abundant in the outer membrane of gram-negative bacteria, can play a role in the reduced effectiveness of many antibiotics [1]. The fact that AMPs’ modes of action include interaction with many different targets on the microbial envelope makes the development of resistance against them complicated. Therefore, AMPs are considered as a convenient base from which to design new antibiotics in order to combat resistance. Currently, antimicrobial peptides are being actively studied. This is shown, for example, by the fact that during the last year, the number of entries in the DBAASP database [2] increased by 1500.

Despite this, antimicrobial peptides are quite poorly used in clinical practice. There are three main reasons that prevent their active use as antibiotics. First, before they can express their full potential antimicrobial activity, peptides can be degraded by proteases (either host or microbial). Secondly, many AMPs are toxic to mammalian cells; finally, they are expensive to produce. 

Although AMPs stand in front of the aforementioned issues, their design, synthesis, and attempts to use them as antibiotics continue: more than 75% of peptides in DBAASP are synthetic, and in the last years, the number of peptides which are at different stages of clinical trials has also increased [3].

Various methods have been used to design new AMPs with high antimicrobial activity, resistance to proteolysis, and low toxicity [4]. These approaches include experimental and computational methods such as mutation-based empirical methods [5,6], statistically-based bioinformatics methods [7,8,9,10], and mechanism-based methods, which include MD simulations [11,12,13,14] and biophysical experiments such as NMR [15,16,17]. One of the best approaches to designing new AMPs is to use bioinformatics methods which are simple, fast, and cost effective. Many of them are based on prediction models (tools) which have been constructed on the basis of the statistical processing of data obtained from different AMP databases. These tools employ different machine learning and data analysis approaches [18,19,20,21,22,23,24,25,26,27,28,29,30,31,32,33,34,35,36,37,38,39,40,41,42,43].

Despite the large number of methods, one of the main obstacles in the design of new peptides is the lack of effective predictive models which are capable of showing high performance when designing new amino acid sequences with high therapeutic effects against particular bacterial strains [44,45].

We developed a tool for AMP prediction, entitled Special Prediction (SP) (https://dbaasp.org/prediction). SP predicts antimicrobial activity based on the mechanisms of action of AMPs against a particular strain, and makes it possible to design and synthesize certain types of peptides with specific properties (for instance, without hemolytic or cytotoxic activity, etc.). Initially, the predictive model was developed for anti-*Escherichia coli ATCC 25,922* peptides [44]. Recently, predictive models for peptides showing potency against *Staphylococcus aureus ATCC 25923*, *Bacillus subtilis* and *red blood cells* (hemolytic activity) have also been developed (see special prediction page of DBAASP https://dbaasp.org/prediction).

In this work, SP is used for the task-oriented design of new AMPs with high therapeutic indexes, meaning that the peptides having high antimicrobial activity against *Escherichia coli ATCC 25922*, should also have low hemolytic activity. (We define therapeutic index here as selectivity index (SI), which was calculated as *LC_10_/MIC*.) The corresponding peptides were synthesized and tested in vitro for activity against *Escherichia coli ATCC 25922*, for hemolytic and cytotoxic activity, and for proteolytic stability. Several peptides were also tested against other gram-negative bacteria (including drug-resistant strains). In order to investigate the mechanisms of action of two special peptides, fluorescence microscopy was also carried out in order to assess their bacterial membrane penetrative properties. The results gained from susceptibility testing have justified the use of the SP tool in the design of new AMPs.

## 2. Results

### 2.1. Selecting the Length for De Novo Design

At the first stage, only short peptides with lengths of 13 aa were designed. There are several reasons for this.

**Figure 1:**
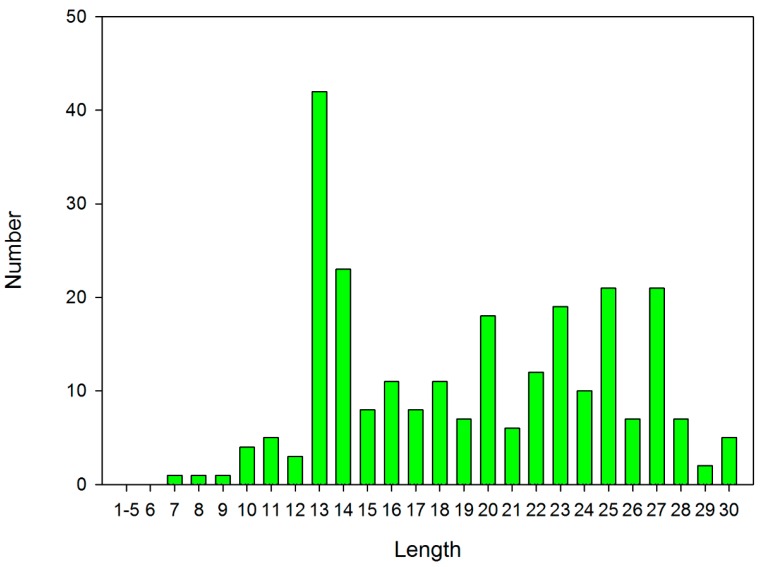
Distribution of length of ribosomal peptides active against *Escherichia coli ATCC 25922*.

Firstly, short peptides have a lower cost, which is important, since high cost is one of the factors that prevents the use of AMPs in clinical practice. Secondly, the length of 13 amino acids (aa) was chosen on the basis of the fact that most short, natural (ribosomal) peptides have a length of 13aa. This is evident from the distribution of peptide lengths which are active against *Escherichia coli ATCC 25922* in the DBAASP database (see Figure 1). Thirdly, 13 aa long peptides have enough resources to adopt the alpha-helical structure, which is crucial for the activity of many AMPs.

### 2.2. SP Models Used for the Design of Peptides

The SP tool predicts whether peptides are active against certain bacterial strains, and whether they have hemolytic activity. The prediction is made on the basis of the clustering of physico-chemical characteristics of peptides, using a semi-supervised machine-learning approach. Algorithm description and a predictive model for anti—*Escherichia coli ATCC 25922* peptides were previously described in [44]. The following 9 characteristics were used: Normalized Hydrophobic moment (M), Normalized Hydrophobicity (H), Charge (C), Isoelectric Point (I), Penetration Depth (D), Orientation of Peptides relative to the surface of membrane (Tilt angle) (O), Propensity to Disordering (R), Linear Moment (L), and In vitro aggregation (A) [46]. Three clusters of peptides with similar characteristics (E1, E2 and E3) were revealed by clustering process on the training set of AMPs. The predictive model was established based on the obtained clusters. The overall results of the prediction on the test set, performed on the basis of a developed predictive model, were: sensitivity (SN) = 0.74, specificity (SP) = 0.85, accuracy (AC) = 0.79, positive predictive value (PPV) = 0.83. It is worth noting that from 140 peptides of positive training set, 85 were grouped into cluster E1, 15 peptides into E2 and 17 into E3, while for the test set, the number of peptides appearing in the E2 and E3 was dramatically less. Consequently, we can say that there is not enough data to correctly assess the PPV of prediction for the models relying on the clusters E2 and E3. This means the predictions on the basis of cluster E1 can be considered reliable, while assessments of reliability of predictions on the basis of E2 and E3 require additional data. For details of the prediction results for *Escherichia coli ATCC 25922*, we recommend consulting research paper [44].

**Figure 2:**
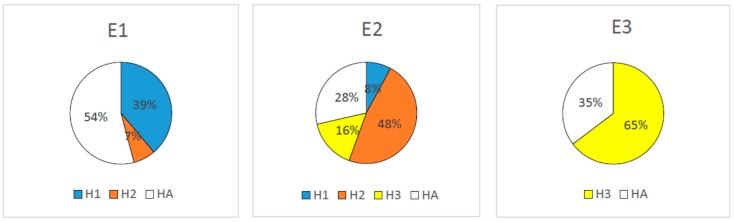
Distribution of peptides, active against *Escherichia coli ATCC 25922* by clusters established for non-hemolytic peptides. E1, E2, and E3—clusters revealed for peptides, active against *Escherichia coli ATCC 25922*; H1, H2, H3—clusters revealed for non-hemolytic peptides; HA—array of peptides which do not belong to the non-hemolytic clusters.

A predictive model for the hemolytic activity of peptides was developed by the same algorithm [44]. The mean values and standard deviations of computed parameters for optimized clusters are given in Table 1. The results of in silico predictions of hemolytic potency of peptides are presented in Table 2. Figure 2 shows how peptides which are active against *Escherichia coli ATCC 25922* are distributed by clusters, which are revealed for non-hemolytic peptides.

### 2.3. Peptide Design

The aim of this work is to design peptides with high antimicrobial activity and low hemolytic activity with the help of SP tools. SP algorithm [44] separates peptides into clusters according to their physico-chemical features, and the necessary requirement for these clusters is to be representative and statistically significant. This might be hard to achieve, due to the paucity of experimental data for some pathogenic organisms. In our studies, novel peptide sequences were generated only for clusters having the most reliable assessments, i.e., for E1. In physicochemical spaces E1 and H1, the most overlapping clusters occur (Figure 2). The value of PPV for Clusters E1 and H1 for test sets is quite high (0.88 for both clusters); therefore, we can state that the data from the Clusters E1 and H1 are appropriate to design non-hemolytic peptides active against *Escherichia coli ATCC 25922*. With the currently available information, we were able to design a predictive model of activity against *Escherichia coli ATCC 25922* bacteria, with additional requirement for low hemolytic activity. A simple algorithm (DSP) was developed for the design of de novo AMPs according to the suggested model (see Methods section for details). Based on DSP, 14 novel peptides were designed and tested. Using the results of experimental testing of these peptides, an additional 3 peptides were manually designed and tested (see below).

### 2.4. Hemolytic Activity of the Designed Peptides

The results of hemolytic activity experiments show that all peptides have hemolytic activity below 10% at a concentration of 50 µg/mL, and most peptides do not have a hemolytic activity at a concentration of 100 µg/mL. Peptide SP 14 has high antimicrobial activity, but hemolytic activity at a concentration of >50 µg/mL is higher than 10%. We suspected that this was due to SP14 high hydrophobicity, and it is known that hydrophobicity in peptides can correlate with hemolytic activity. We decided to remove C-terminal isoleucine residue in order to decrease the overall hydrophobicity of the peptide, and we repeated in vitro testing of the remaining 12 residue peptide (SP15 in Table 3. This strategy worked, as the peptide SP15 retained antimicrobial activity, while not exhibiting hemolytic activity at a concentration of 100 µg/mL.

### 2.5. Analysis of the Results of In Vitro Tested Peptides

The data for the development of the predictive model were obtained from susceptibility test experiments where bacterial growth was performed in a broth poor with salt (NaCl). 

At the same time, there are data suggesting that MICs for cationic peptides depend on NaCl concentrations [20], so we decided to test peptides in two different mediums: in Luria Bertani (LB) medium supplemented with NaCl and in LB medium, not supplemented with NaCl (Table 3). From 15 peptides predicted by the SP tool as being active against *Escherichia coli ATCC 25922* and tested in vitro in LB medium without NaCl, 14 were active (MIC < 50), meaning that the SP tool correctly predicted biological activity in 93% of cases. In a LB medium containing NaCl, 12 peptides showed antimicrobial potency, corresponding to 80% correct predictions.

The peptides SP1-SP4 were tested against 16 other gram-negative bacterial strains in the Departments of Pathology Medicine/Infectious Diseases, University of Texas Health Science Center, at San Antonio. These results are presented in Table 4. All peptides demonstrated high antimicrobial activity against 8 strains from the 16 tested. As the control, Meropenem, which is one of the most effective antibiotics, especially against Gram-negative organisms [47], was used. Importantly, our peptides were active even against drug-resistant *P. aeruginosa* J4228 (Meropenem Resistant), *A. baumannii* Josh 28, and *Escherichia coli ARLG-1012* (NDM Resistant).

### 2.6. Cytotoxicity of the Designed AMPs

**Figure 3:**
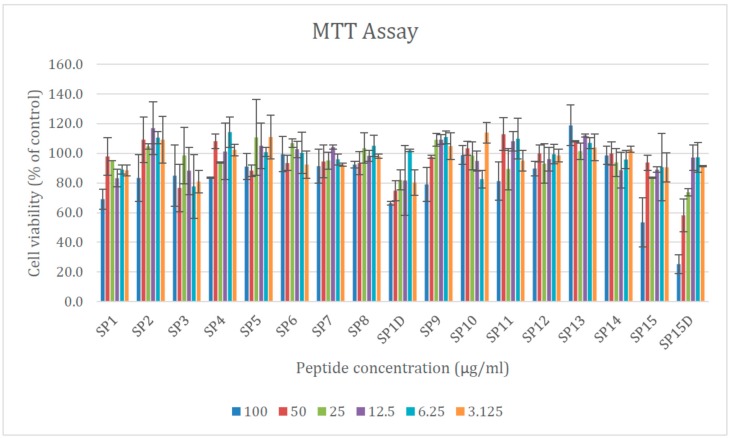
Viability of the cells after being treated with the peptides at different concentrations.

Cytotoxicity of in silico designed peptides was measured against Log phase Hepa 1-6 cells using the 3-(4, 5-dimethylthiazol-2-yl)-2, 5- diphenyltetrazolium bromide (MTT) assay. The corresponding results show that for all peptides, the viability of a cell is high at peptide concentrations close to MIC (Figure 3).

### 2.7. The Proteolytic Stability of the Synthesized AMPs

As mentioned above, AMPs may be digested by the microbial or host proteases. Resistance of peptides to proteases is an essential requirement for peptide-based drug design. To assess the resistance of peptides against proteases, peptides were exposed to Proteinase K and α-chymotrypsin. It can be seen that all tested L-peptides are fully or partially digested by the proteases (Table 3). It is known that the proteolytic stability of the peptide may be increased if modified amino acids are used; for example, D-amino acids instead of L-. For this purpose, 2 peptides were synthesized with the D-amino acids – SP1D and SP15D. These peptides were tested for antimicrobial activity against *Escherichia coli ATCC 25922*, hemolytic activity and proteolytic stability. The results show that peptides consisting of D-amino acids are not digested by proteases. The antimicrobial activity of peptides SP1D and SP1 towards *Escherichia coli ATCC 25,922* are almost the same; however, peptide SP15D has higher antimicrobial activity than its enantiomer (SP15). In addition, the SP1D peptide exhibits a certain hemolytic activity at concentration of 50 µg/mL (15% hemolysis), while SP15D does not show hemolytic activity at 100 µg/mL. We must also note that SP15D has one of the lowest values of MIC among all 1333 short (<17aa) anti—*Escherichia coli ATCC 25922* peptides obtained from DBAASP at the time of the article submission (Figure S1). It must be noted that this peptide, as well as the other designed peptides, is unique and cannot be found in any peptide database. So, we can consider this peptide as a suitable candidate for further design to create new antimicrobial drugs.

### 2.8. Investigation of Permeability of the Bacterial Membrane for FITC Dye by Fluorescence Microscopy

**Figure 4:**
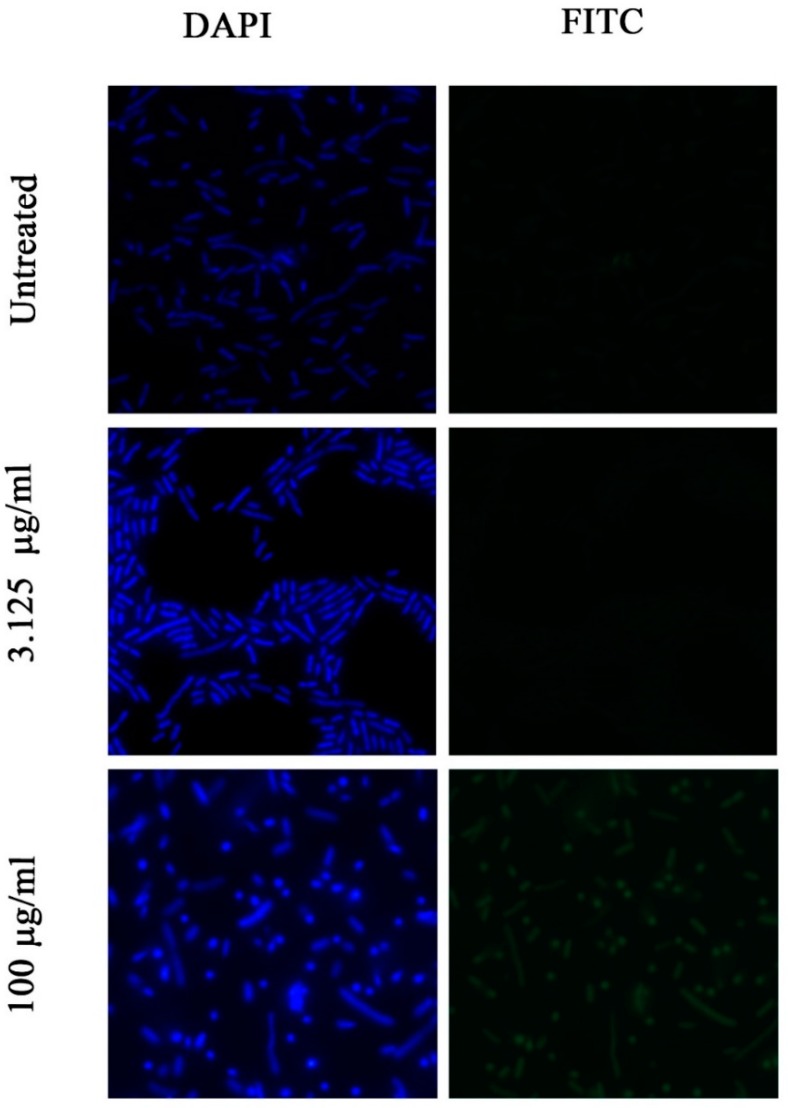
Images of fluorescence of DAPI a FITC before and after treatment of SP15 with concentrations of 100 µg/mL and close to MIC.

**Figure 5:**
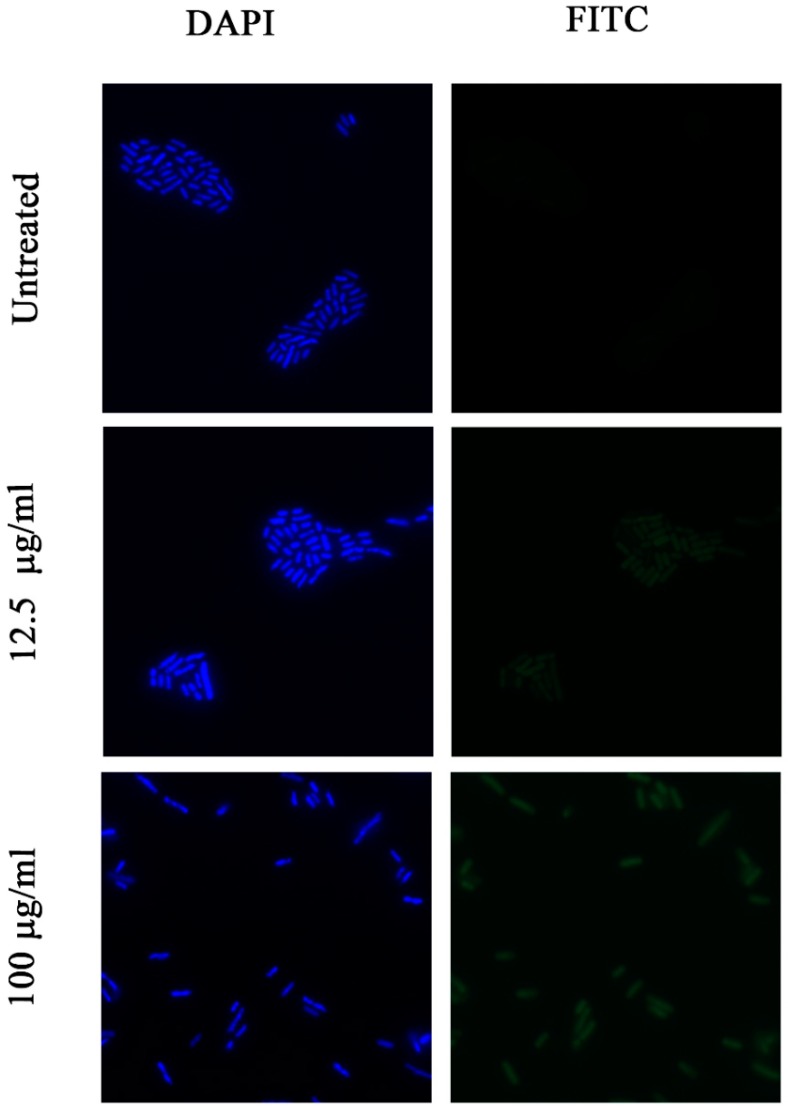
Images of fluorescence of DAPI and FITC before and after treatment of SP4 with concentrations of 100 µg/mL and close to MIC.

The mechanisms of action of AMPs are still being studied. It is known that most AMPs act on the envelope of bacteria, but it is not fully understood how they interact with the cytoplasmic membranes. We decided to study the permeability of the membrane after interaction with de novo designed peptides by staining them with FITC. It should be emphasized that FITC is unable to traverse the intact cytoplasmic membrane, but if the peptide has affected the membrane, the permeability of the latter should be increased. On the other hand, for peptides with high penetration abilities, acting inside the cytoplasmic membrane, we expected no changes in the permeability of membrane. We selected two peptides from the designed peptide set, with the highest (SP15) and lowest (SP4) value of their penetration abilities, which were determined according to CPPpred predictions (http://distilldeep.ucd.ie/CPPpred/) [48]. Bacterial cells were exposed to FITC after their treatment with the peptides at two concentrations: 100 µg/mL and 3.125 µg/mL for SP15, and 100 and 12.5 µg/mL for SP4. The fluorescence was compared with untreated bacterial cells. (Figure 4 and Figure 5).

After the addition of the PFA to the cells treated by peptides at a concentration of 100 μg/mL, the permeability of the membrane for FITC increased, as compared to the control. But, at the concentrations close to MIC, bacteria treated with SP15 did not show any visible differences in FITC fluorescence from control. Despite this, for SP4, at MIC concentration (25 µg/mL), low FITC fluorescence is visible. We concluded that at a concentration of 100 μg/mL, both peptides make significant changes in the membrane structure, and that the membrane becomes permeable for FITC. At the same time, at concentrations close to MIC (3.125 µg/mL), SP15 does not change the membrane structure (it doesn’t make it permeable for FITC), while SP4 changes it and makes it FITC- permeable. Therefore, we suggest that SP15 acts differently at close to MIC and higher concentrations: at MIC it penetrates the membrane without disrupting it and apparently acts on intracellular targets, while at higher concentrations, it causes significant changes in the membrane structure. It should be noted that FITC fluorescence is visible only after fixation of bacterial cells with PFA. So, the corresponding changes in the membrane structure are likely to be dynamic and can be restored (see Section 5.10).

## 3. Discussion

We developed and experimentally-tested a new predictive tool (SP) to design novel bioactive peptides with a high therapeutic index. SP predictions are based on user-driven analyses of known peptides possessing biological activity against a chosen class of bacterial pathogens. The biological activity of peptides is predicted based on their calculated physicochemical features. Clustering of the peptides active against a particular organism (strain) allows us to assume that peptides, having similar physico-chemical properties and united into the same clusters, have similar mechanisms of actions against the target organism. So, we hoped to be able to predic, with the SP tool, not only the antimicrobial potency of peptides, but also the mechanism of their action, and accordingly, to gain the ability to design peptides with certain mechanisms of actions. Although there are various data about the mechanisms of action of antimicrobial peptides, many aspects of the mechanisms are not known. According to the Shai-Matsuzaki-Huang (SMH) model [49,50,51], most AMPs interact with the membrane, causing morphological changes in the membrane structure. Most known mechanisms of action of antimicrobial peptides can be attributed to three types: 1) peptides that interact with bacterial membrane and destroy it [52,53,54]; 2) peptides altering the structure of the local microdomains of the membrane, thus causing disturbances of the metabolic processes associated with it; 3) peptides penetrating the membrane and acting on intracellular targets (inhibiting the synthesis of macromolecules, the metabolic/enzymatic functions [55] and a cell-wall/membrane formation [56]). Peptides of the first type can act by the barrel-stave [57], carpet [58], toroidal pore or wormhole [59] and the aggregation mechanism models [60]. Antimicrobial peptides with different known mechanisms of action were clustered into 3 groups (P1, P2, P3) [61]. Peptides falling into the the P1 and P3 groups are pore-forming (interacting with the membrane and destroying it). Peptides from P2 penetrate the membrane without destroying it. Consequently, according to the results of interactions with the membrane, we revealed two types of peptides: membrane destroying (P1 and P3) and cell penetrating (P2). It is worth noting that most peptides from the P2 group contain a large amount of Pro and Arg, and sometimes aromatic amino acids, especially Trp. Taking into consideration the recent data, the last classification could be considered as conditional. The recent data show that the same peptide can act by different mechanisms depending on the time of its action [62]. The authors of [62] show that the synthetic peptide PuroA (Arg and Trp rich peptide) first penetrates the membrane and binds to nuclear acids without disrupting the cell membrane integrity, and after 40-45 min, creates membrane pores. We have compared the results of clustering, obtained by us, to the grouping of AMP according to [61]. This comparison shows that both cell-penetrating and pore-forming peptides fall into the E1 cluster. If we suggested that Puro A’s features are shared features of AMP, our results adequately reflect the reality. But it is more reasonable to think that there are purely penetrating or pore-forming peptides, as well as peptides like Puro A. If this is true, we can say that at approximation, when physico-chemical properties of peptides are described by characteristics relying on hydrophobicity and charge, it is not possible to reveal any essential differences between cell penetrating and pore-forming peptides. In any case, it is possible to look for the characteristics which can discriminate pore-forming peptides from penetrating, especially if we take into consideration a well-defined peculiarity of the amino acid composition of cell-penetrating peptides. So, the physico-chemical properties used in this work characterize shared features of active peptides and reveal a group of peptides with particular features requires taking additional characteristics into consideration. So, the search for new characteristics should be the task of future work. For example, as mentioned, cell penetrating peptides are rich in Arg, Trp and Pro. It is supposed that cation-π interactions make a major contribution to the penetration capabilities of peptides [63]. Our description of the physico-chemical features of peptides did not take into consideration differences in the side chains of Arg and Lys and specificities of their interactions with Trp. So, we suppose that if we take into account cation-π interactions, the discrimination of cell penetrating peptides from pore-forming will become possible.

It is worth noting that the degree of total effect of AMPs on the membrane structure depends on the concentration of the peptide; therefore, peptides can act by different mechanisms depending on their concentration [64]. Our results of fluorescence microscopy studies of bacterial membrane permeability are in agreement with these suggestions. Our data on the investigation of FITC penetration into *Escherichia coli ATCC 25,922* bacterial cells, treated by SP15, show that at the MIC concentration, the peptide does not disrupt the membrane, but at high concentrations, FITC penetration rises as a result of the membrane disruption. 

Thus, it can be said that the existing features of the AMPs complicate the possibility of predicting the mechanisms of their action on the basis of sequence-dependent characteristics such as hydrophobicity and charge. Nevertheless, the further development of a SP tool will make it possible to predict the mechanisms of action for at least some groups of antimicrobial peptides which may act by single mechanisms.

## 4. Conclusions

Seventeen peptides were predicted to have antimicrobial activity against *Escherichia coli ATCC 25922.* After in vitro testing, it was demonstrated that almost all of the peptides had high antimicrobial potency against *Escherichia coli ATCC 25922*, and did not exhibit hemolytic and cytotoxic effects. However, these peptides can still be digested by the proteases. Replacement L- to D- amino acids in case of 2 peptides (SP1D and SP15D) greatly increased the stability of peptides to the protease digestion. One of these peptides, SP15D, has one of the lowest MICs among all known short (less than 17 residues) anti *Escherichia coli ATCC 25,922* peptides. An assessment of the membrane penetrative ability of de novo designed SP4 and SP15 peptides allowed us to conclude that at high concentrations, both peptides change membrane morphology, but at concentrations close to MIC, they behave differently: SP4 significantly affects membrane structure, while SP15 does not. Susceptibility testing against a panel of gram-negative bacteria (including drug-resistant strains) for de novo designed peptides demonstrated high antimicrobial potency against clinically important *P. aerogenosa* and *A. baumannii* pathogens.

## 5. Methods

### 5.1. Predictive Models

Peptide design was carried out on the basis of predictive models of the activity of peptides against *Escherichia coli ATCC 25922* and human erythrocytes. The algorithm for predictive model development is described in detail in [44]. The model is based on the clustering of physico-chemical characteristics of peptides using a semi-supervised machine-learning approach, relying on density-based clustering algorithm DBSCAN [65]. The following 9 features were used in the QSAR study: Normalized Hydrophobic moment (M), Normalized Hydrophobicity (H), Charge (C), Isoelectric Point (I), Penetration Depth (D), Orientation of Peptides relative to the surface of membrane (Tilt angle) (O), Propensity to Disordering (R), Linear Moment (L), and In vitro aggregation (A). Hydrophobic scale, described by Moon and Fleming [66], was used for the definition of Hydrophobic moment, Hydrophobicity and Linear Moment. Penetration Depth and Tilt angle was defined by the method described in the paper [67]. Propensity to Disordering was calculated by Uversky’s formula [68]. The detailed definitions of these characteristics can be found in the paper of Vishnepolsky and Pirtskhalava [46]. Due to the presence of the structure-dependent peptide characteristics (Hydrophobic moment, Penetration Depth, Tilt angle), these characteristics were calculated in the α-helical approximation, energetically the most favorable conformation in the membrane environment [69].

To generate a training and test set for the development of predictive models of non-hemolytically active peptides, 10–35 residue long sequences were used. This is an optimal length interval for which a statistically reliable model might be built. But in this case, the problem was the correct assessment of hydrophobic moments for the peptides with lengths longer than 24 residues. To calculate the hydrophobic moment of the peptides with length >24 aa, a sliding window was used. Twenty-four aa was selected as an optimal window length [46]. The fragment with a maximum value of the hydrophobic moment was characterized a whole peptide. The same fragment was used to calculate other structure-dependent characteristics such as Penetration Depth and Tilt angle. Non-structure-dependent characteristics were assessed for the full sequence of peptides.

A positive set corresponded to the peptides that were not-active against human erythrocytes, while a negative set corresponded to hemolytic peptides. A peptide was defined as hemolytic if, at the concentration <40 μg/mL of this peptides, a lysis of >40% of erythrocytes was detected. Peptides, defined as non-active in the DBAASP were included in the positive set. After satisfying the conditions of non-redundancy and excluding peptides with D-amino acids, the positive and negative training sets contained 120 sequences each, whilst positive and negative test sets had 43 sequences each.

### 5.2. Evaluation of the Quality of the Prediction and Definition of the Therapeutic Index

The following equations were used to evaluate the quality of the prediction:

*S*N = *TP*/(*TP* + *FN*)

*S*P = *TN*/(*TN* + *FP*)

*AC* = (*TP* + *TN*)/(*TP* + *FN* + *TN* + *FP*)

*BAC* = (*S*P+ *SN*)/2

*PPV* = *TP*/(*TP* + *FP*)

where *SN* is sensitivity, *SP* is specificity, *AC* is accuracy, *BAC* is balance accuracy, *PPV* is positive predictive value, *TP* is true positive, *TN* is true negative, *FP* is false positive, and *FN* is true negative

Therapeutic index (TI) is defined as selectivity index (SI), and it is calculated as follows:

*TI*(*SI*) = *LC*_10_/*MIC*

where *LC*_10_ is Lethal Concentration of the AMPs which kills 10% of the human erythrocytes, MIC is minimum inhibitory concentration.

### 5.3. Peptide Design

The most statistically reliable clusters were used to generate new amino acid sequences for in vitro testing against gram-negative bacteria.

A simple algorithm named DSP, based on the SP model of prediction, was developed for the design of de novo AMPs. According to the algorithm, four steps of action are suggested to create new sequences with antimicrobial potency while also being non-toxic to red blood cells. In the first stage, 13 aa long sequences were generated using a random number generator. Amino acid frequencies used for sequence generation correspond to frequencies in the set of linear, ribosomal AMPs. The set was formed on the basis of DBAASP data (https://dbaasp.org/statistics), and frequencies are given in the Table S1. In the second stage, from among randomly generated sequences were selected those that were active against Escherichia coli ATCC 25922 according to SP. At the third stage, from among sequences active against Escherichia coli were chosen non-toxic (not active against Human Erythrocytes) ones, again using SP algorithm. At the end (fourth stage), the sequences selected in the previous three steps were checked in the following databases: Uniprot [70], DBAASP [2], APD [71], CAMP [72], DRAMP [73]). It was interesting to know which part of the randomly-generated sequences met the aforementioned requirements. Consequently, 5 10^5^ sequences were generated at the first stage, and 48 121 sequences remained after the fourth stage. Fourteen sequences were randomly selected from the remaining sequences to perform in vitro testing of the SP prediction model. On the basis of these sequences, 14 peptides were synthesized and tested in vitro on Escherichia coli ATCC 25922 susceptibility, on hemolytic/Cytotoxic activity, and stability against proteases. Using the results of the experimental testing of these 14 peptides, an additional 3 peptides were manually designed and tested (see Section 2.4 and Section 2.7).

### 5.4. Peptide Synthesis

The designed peptides were synthesized by LifeTein^®^ LLC (South Plainfield, NJ, USA) using PeptideSynTM technology. All peptides have C-terminal amides. Peptides were delivered as salts of hydrochloride. The purity of the peptides was determined by high-performance liquid chromatography (HPLC) and was >98%.

### 5.5. Susceptibility Testing against Escherichia coli ATCC 25922

The MICs of the designed cationic peptides against *Escherichia coli ATCC 25,922* strains were determined by the broth microdilution method, as described by Hancock et al. [74]. All MIC evaluations were performed using Luria Bertani (LB) medium. Briefly, a range of each peptide concentrations (100 µg/mL, 50 µg/mL, 25 µg/mL, 12.5 µg/mL, 6.25 µg/mL, 3.125 µg/mL) was prepared by serial dilution in NaCl-depleted LB medium and added to an equal volume (250 µl) of exponentially grown bacterial culture, so that final bacterial suspension contained 5 × 10^5^ CFU/mL. The samples were incubated in 2 mL polypropylene microtubes at 37 °C overnight at 300 rpm. The MIC was defined as the concentration at which no microbial growth was detected spectrophotometrically, via readings of optical density (OD) at 600 nm (Biotek ELX800 microplate reader). Growth medium containing only bacterial cells was used as a negative control. Each MIC test was carried out in two replicates and repeated three times.

### 5.6. Susceptibility Testing against other Different Gram-Negative Bacterial Strains

The following strains were used: *P. aeruginosa* (ATCC 27,853 and resistant strains: J4228 (R: Meropenem), BB2013-100 (FQR)); *A. baumannii* (Josh 28 and resistant strain Josh 230 (OXA-48); *E. cloacae* (resistant strains: BB2012-181(R: Meropenem), BB2013-32 (FQR)); *E. aerogenes* (resistant strain St. L P63 (NDM-1), *E. asburiae/cloacae* (resistant strain St. L P23 (NDM-1), *K. pneumonia* (J3702 and resistant strains: BB2009-209 (KPC-2), Oschner KP-1), *Escherichia coli* (BW25113 (7636) and resistant strains: JW55034 (11430) (Tol neg), BB2013-30 (R:carbapenem), ARLG-1012 (NDM)). Susceptibility testing was performed using non-cation adjusted MHB broth and polypropylene plates. Testing was also done in the presence and absence of acetic acid and BSA. For comparisons, meropenem (class of carbapenems), which is more effective against Gram-negative organisms [47], and the standard CLSI method, were used.

### 5.7. Hemolytic Activity Assessment

Fresh Human Blood (500 µl) was collected into 2 mL microcentrifuge tubes containing heparin (30 units) and immediately centrifuged at 3700× *g* rpm for 5 min at room temperature. The pellet containing erythrocytes was washed 3 × 5 min in 1x PBS at 3700 rpm, RT. Finally, the pellet containing red blood cells (RBC) was diluted with 1x PBS to obtain 2% RBC suspension and added to an equal amount (250 µL) of serially-diluted (100 µg/mL, 50 µg/mL, 25 µg/mL, 12.5 µg/mL, 6.25 µg/mL) AMPs to be tested. The microtubes were incubated at 37 °C for 1 h. The samples were centrifuged at 3700× *g* rpm for 5 min and the supernatant (200 µL) was taken from each microtube and added to a LC6-well microplate for the absorption analysis on microplate reader at 450 nm. The positive control (100% hemolysis) consisted of 0.1% Triton x-100 in 1xPBS (pH 7.4), while 1xPBS served as a negative control (0% hemolysis). Each hemolysis activity assessment test was carried out in two replicates and repeated three times.

### 5.8. Cytotoxicity Assay of the Antimicrobial Peptides

Cytotoxicity of in silico designed LCAPs was measured using the 3-(4,5-dimethylthiazol-2-yl)-2,5-diphenyltetrazolium bromide (MTT) assay. Log phase Hepa 1–6 cells were seeded onto a 96-well cell-culture plate at 10 × 10^5^ per well. The cells were incubated for 24 h at 37 °C under 5% CO_2_.

A solution of peptides in DMEM was added to the wells at final concentrations of 100, 50, 25, 12.5, 6.25 and 3.125 µg/mL. The cells were incubated for 24 h at 37 °C under 5% CO2. After removal of the medium, a solution of 0.5 mg/mL MTT (100 µL/well) was added for an additional 4 h incubation, allowing viable cells to reduce the yellow tetrazolium salt (MTT) into dark-blue formazan crystals. After removal of the medium, formazan extraction was performed with 100 µL DMSO, and the amount of formazan was determined to measure the absorbance value (OD490) in a plate reader (BioTek ELx800). The following formula was used to calculate cell viability: Viability (%) = (mean Absorbance value of treatment group/mean Absorbance value of control) × 100.

### 5.9. The Proteolytic Stability towards α-Chymotrypsin and Proteinase K Digestion

To test whether the peptides were digested by proteases, 10 μg of each peptide was incubated with, α-chymotrypsin or Proteinase K at a molar ratio of 1000 :1, 500 : 1 (peptide:enzyme), respectively, in the digestion buffer (50 mM Tris–HCl, pH 7.4, 5 mM CaCl_2_) at 37 °C for 16 h. The reaction was stopped by incubating the samples at 95 °C for 3 min. The digestion mixture was sampled and analyzed by 16.5% Tris-Tricine–SDS–PAGE. The gels were stained with 0.15% Coomassie Brilliant Blue G 250.

Peptide degradation was considered positive if the peptide bands disappeared from the gel after protease treatment.

### 5.10. Assessment of Membrane Penetrating Properties of Antimicrobial Peptides by Fluorescence Microscopy

Permeabilization of the membrane of *Escherichia coli ATCC 25922*, induced by the antimicrobial peptide, was visualized by green fluorescent dye FITC, which is unable to traverse the cytoplasmic membrane of cells unless it has been permeabilized by a peptide. The O/N culture of *Escherichia coli ATCC 25922* (approximately 4 × 10^7^ cells in 100 mL) were exposed to antimicrobial peptides at a final concentration of 100 mg/mL, at 37 °C for 60 min. After incubation, peptides were removed by centrifugation at 5500× *g* rpm for 5 min and bacterial cells were fixed with 4% PFA at 37 °C for 20 min. To remove PFA, the samples were washed twice with 10 mM sodium phosphate buffer, (NaPB) pH 7.4. Only after treating with PFA was it revealed that at a certain concentration of peptides, membranes became permeable for FITC. As an explanation for this fact, we suggest that destructive peptides form temporary defects in the membrane; if not fixed, this defect can be repaired. Thus, in order to assess whether the peptides have damaged the bacterial membrane, it is necessary to fix bacterial cells by PFA.

Finally, the pellet was resuspended in 10 mM NaPB containing FITC (6 μg/mL) and incubated at RT for 25 min. To remove FITC, the samples were washed twice with 10 mM (NaPB) and the pellets were resuspended in DAPI (5 μg/mL)/10 mM NaPB and left for incubation at RT for 15 min. The samples were washed with 10 mM NaPB and the pellet was resuspended in 10 mM NaPB. The cell suspension was poured on to poly(L-lysine)-coated coverslips placed in petri dishes and kept at 30 °C for 45 min to allow adhesion to the glass slides to occur. After incubation, coverslips were placed onto microscope slides and sealed with nail polish. The slides were then examined under BX 41 Olimpus fluorescence microscope, equipped with an oil-immersion objective (×100) and captured with an Olimpus Q-Color5 CCD camera.

## Figures and Tables

**Table 1:** Average values and standard deviation of 9 physico-chemical characteristics for optimized clusters for 10-16 aa long peptides non-active against Human erythrocytes.

Mean Values ± SD of Attributes	Cluster H1 (MHCIORLS *^a^*)	Cluster H2 (MA *^a^*)	Cluster H3 (MCIA *^a^*)
M ± σ	**1.42 ± 0.37**	**0.33 ± 0.13**	**1.07 ± 0.24**
H ± σ	**0.09 ± 0.56**	−0.14 ± 0.73	−0.71 ± 0.31
C ± σ	**6.9 ± 1.93**	3.87 ± 3.12	**2.64 ± 0.79**
I ± σ	**13.75 ± 0.58**	10.73 ± 2.51	**10.81 ± 0.48**
D ± σ	16.74 ± 4.2	23.9 ± 6.39	15 ± 4.79
O ± σ	**100.76 ± 26.29**	82.42 ± 43.36	95.96 ± 28.04
R ± σ	**−0.32 ± 0.29**	−0.26 ± 0.24	0.19 ± 0.22
L ± σ	**0.31 ± 0.08**	0.34 ± 0.1	0.32 ± 0.08
A ± σ	2.25 ± 10.54	**2.68 ± 7.27**	**13.05 ± 16.16**
S ± σ	**13.84 ± 3.35**	17.39 ± 6.09	16.72 ± 4

*^a^* Attributes, which characterize space where cluster was formed. Mean and SD of these attributes are marked in bold.

**Table 2:** Results of not hemolytic peptide prediction on the training and test sets.

		*TP*	*TP + FN*	*FP*	*TN + FP*	*S_n_*	*S_p_*	*AC*	*PPV*
Training Set	Cluster H1	50	120	13	120				0.79
	Cluster H2	31	120	4	120				0.89
	Cluster H3	25	120	8	120				0.76
	**All Clusters**	**106**	**120**	**25**	**120**	**0.88**	**0.79**	**0.84**	**0.81**
	Cluster H1	14	43	2	43				0.88
Test Set	Cluster H2	11	43	3	43				0.79
	Cluster H3	7	43	5	43				0.58
	**All Clusters**	**32**	**43**	**10**	**43**	**0.74**	**0.77**	**0.76**	**0.75**

**Table 3:** Assessments of susceptibility of *Escherichia coli ATCC 25922* (MIC), hemolytic activity (LC_10_) proteolytic stability (STP) and therapeutic index (TI) for the designed peptides.

Name	Sequence	MIC (µg/mL)		STP (Peptide/Protease M Ratio)				LC_10_ (µg/mL)	TI **
		At NaCl	Without NaCl	Proteinase K		α-chymotrypsin			
				1000:1 Ratio	500:1 Ratio	1000:1 Ratio	500:1 Ratio		
SP1	AIKIRKLFKKLLR	12.5–25	3.125–6.25	D	NT	D	NT	>100	>16
SP2	GIKIRKLFKKLLR	6.25–12.5	3.125–6.25	D	NT	D	NT	>100	>16
SP3	GWAKLITKAIKKI	25–50	12.5–25	PD	PD	D	NT	50–100	4
SP4	GIKFFLKKLKKHI	25–50	6.25–12.5	PD	PD	D	NT	>100	>8
SP5	IRPAKLRWFKKIK	>100	12.5–25	D	NT	D	NT	>100	>4
SP6	RLFIKKLKFITRR	25–50	3.125–6.25	PD	D	D	NT	>100	>16
SP7	NAMRGAKRVWRHI	>100	50–100	PD	PD	D	NT	>100	>1
SP8	KFRKFGKQVWVRL	12.5–25	3.125–6.25	PD	D	D	NT	>100	>16
SP1D *	aikirklfkkllr	12.5–25	3.125–6.25	ND	ND	ND	ND	25–50	4–8
SP9	KVWSRLRKIFSTR	6.25–12.5	3.125–6.25	D	NT	D	NT	50–100	8–16
SP10	AKVLKISRRAFRK	>100	25–50	D	NT	D	NT	>100	>2
SP11	IRRWRLHWFRRAI	12.5–25	3.125–6.25	PD	D	D	NT	>100	>16
SP12	IRRRIRLIVRRQI	12.5–25	1.56–3.125	ND	PD	D	NT	>100	>32
SP13	HFKIRKRFVKKLV	>100	6.25–12.5	PD	D	D	NT	>100	>16
SP14	RWIRWVWRKKLRI	12.5–25	3.125–6.25	PD	D	D	NT	50–100	8–16
SP15 *	RWIRWVWRKKLR	3.125–6.25	0.78–1.56	PD	PD	PD	PD	>100	>64
SP15D *	rwirwvwrkklr	0.78–1.56	0.39–0.78	ND	ND	ND	ND	>100	>128

D—Digested; NT—not tested (if a peptide digested by a protease in a lower concentration of the protease, the experiment for the higher concentration was not carried out); PD—Partially digested; ND—not digested; LC_10_ (µg/mL) is a concentration required for 10% hemolysis; STP—Stability Towards Proteases at Peptide to Protease Molar ratio;* peptides, which sequences were manually changed from de novo designed sequences generated by DSP;** TI= LC_10_/max(MIC_without NaCl_).

**Table 4:** In vitro testing of the peptides SP1–SP4 against different gram-negative bacterial strains.

Isolate #	Organism ID	Phenotype	MIC (µg/mL) Meropenem	MIC (µg/mL) SP1	MIC (µg/mL) SP1	MIC (µg/mL) SP3	MIC (µg/mL) SP4
ATCC 27853	*P. aeruginosa*	CLSI Control	1	4	4	8	8
J4228	*P. aeruginosa*	R: Meropenem	>64	8	8	16	16
BB2013-100	*P. aeruginosa*	FQR	1	32	32	32	32
Josh 28	*A. baumannii*	Susceptible	32	4	4	4	2
Josh 230	*A. baumannii*	OXA-48	1	16	16	16	4
BB2012-181	*E. cloacae*	R: Meropenem	16	>32	32	16	>32
BB2013-32	*E. cloacae*	FQR	0.5	>32	>32	16	16
St. L P63	*E. aerogenes*	NDM-1	16	>32	32	16	16
St. L P23	*E. asburiae/cloacae*	NDM-1	16	>32	>32	32	>32
BB2009-209	*K. pneumoniae*	KPC-2	32	>32	>32	>32	>32
J3702	*K. pneumoniae*	Susceptible	≤0.125	>32	>32	16	32
Oschner KP-1	*K. pneumoniae*	KPC-3	16	>32	>32	>32	>32
BW25113 (7636)	*E. coli*	WT, Tol parent strain	≤0.125	8	8	4	8
JW55034 (11430)	*E. coli*	Tol neg	≤0.125	8	8	4	8
BB2013-30	*E. coli*	R:carbapenem	1	16	16	8	16
ARLG-1012	*E. coli*	NDM	64	8	8	4	8

## Supplementary Materials

The following are available online at https://www.mdpi.com/1424-8247/12/2/82/s1. Figure S1: Screenshot of DBAASP Ranking Search Page. Table S1: Amino acid distribution used in the sequence generation algorithm.

## References

[1] Pagès J.M., Masi M., Barbe J. (2005). Inhibitors of efflux pumps in Gram-negative bacteria. Trends Mol. Med..

[2] Pirtskhalava M., Gabrielian A., Cruz P., Griggs H.L., Squires R.B., Hurt D.E., Grigolava M., Chubinidze M., Gogoladze G., Vishnepolsky B. (2016). DBAASP v.2: An Enhanced Database of Structure and Antimicrobial/Cytotoxic Activity of Natural and Synthetic Peptides. Nucl. Acids Res..

[3] Greber K.E., Dawgul M. (2017). Antimicrobial Peptides Under Clinical Trials. Curr. Top. Med. Chem..

[4] Li J., Koh J.-J., Liu S., Lakshminarayanan R., Verma C.S., Beuerman R.W. (2017). Membrane Active Antimicrobial Peptides: Translating Mechanistic Insights to Design. Front. Neurosci..

[5] Grieco P., Luca V., Auriemma L., Carotenuto A., Saviello M.R., Campiglia P., Barra D., Novellino E., Mangoni M.L. (2011). Alanine scanning analysis and structure–function relationships of the frog-skin antimicrobial peptide temporin-1Ta. J. Pept. Sci..

[6] Hänchen A., Rausch S., Landmann B., Toti L., Nusser A., Süssmuth R.D. (2013). Alanine scan of the peptide antibiotic feglymycin: Assessment of amino acid side chains contributing to antimicrobial activity. ChemBioChem.

[7] Fjell C.D., Hiss J.A., Hancock R.E., Schneider G. (2011). Designing Antimicrobial Peptides: Form Follows Function. Nat. Rev. Drug Discov..

[8] Torrent M., Di Tommaso P., Pulido D., Nogués M.V., Notredame C., Boix E., Andreu D. (2012). AMPA: An Automated Web Server for Prediction of Protein Antimicrobial Regions. Bioinformatics.

[9] Jenssen H., Lejon T., Hilpert K., Fjell C.D., Cherkasov A., Hancock R.E.W. (2007). Evaluating Different Descriptors for Model Design of Antimicrobial Peptides with Enhanced Activity toward P. aeruginosa. Chem. Biol. Drug. Des..

[10] Taboureau O., Olsen O.H., Nielsen J.D., Raventos D., Mygind P.H., Kristensen H.H. (2006). Design of Novispirin Antimicrobial Peptides by Quantitative Structure-Activity Relationship. Chem. Biol. Drug Des..

[11] Wang Y., Chen C.H., Hu D., Ulmschneider M.B., Ulmschneider J.P. (2016). Spontaneous formation of structurally diverse membrane channel architectures from a single antimicrobial peptide. Nat. Commun..

[12] Li J., Liu S., Koh J.-J., Zou H., Lakshminarayanan R., Bai Y., Pervushin K., Zhou L., Verma C., Beuerman R.W. (2015). A novel fragment based strategy for membrane active antimicrobials against MRSA. Biochim. Biophys. Acta Biomembr..

[13] Li J., Liu S., Lakshminarayanan R., Bai Y., Pervushin K., Verma C., Beuerman R.W. (2013). Molecular simulations suggest how a branched antimicrobial peptide perturbs a bacterial membrane and enhances permeability. Biochim. Biophys. Acta Biomembr..

[14] Fox J.S., Li J., Tan Y.S.N., Nguyen M., Pal A., Ouaray Z., Yadahalli S., Kannan S. (2016). The multifaceted roles of molecular dynamics simulations in drug discovery. Curr. Pharm. Des..

[15] Saravanan R., Li X., Lim K., Mohanram H., Peng L., Mishra B., Basu A., Lee J., Bhattacharjya S., Leong S.S. (2014). Design of short membrane selective antimicrobial peptides containing tryptophan and arginine residues for improved activity, salt-resistance, and biocompatibility. Biotechnol. Bioeng..

[16] Mohanram H., Bhattacharjya S. (2014). b-boomerang antimicrobial and antiendotoxic peptides: Lipidation and disulfide bond effects on activity and structure. Pharmaceuticals.

[17] Jeong J.-H., Kim J.-S., Choi S.-S., Kim Y. (2016). NMR structural studies of antimicrobial peptides: LPcin analogs. Biophys. J..

[18] Jenssen H., Fjell C.D., Cherkasov A., Hancock R.E. (2008). QSAR Modeling and Computer-Aided Design of Antimicrobial Peptides. J. Pept. Sci..

[19] Fjell C.D., Jenssen H., Hilpert K., Cheung W.A., Pante N., Hancock R.E., Cherkasov A. (2009). Identification of Novel Antibacterial Peptides by Chemoinformatics and Machine Learning. J. Med. Chem..

[20] Cherkasov A., Hilpert K., Jenssen H., Fjell C.D., Waldbrook M., Mullaly S.C., Volkmer R., Hancock R.E. (2009). Use of Artificial Intelligence in the Design of Small Peptide Antibiotics Effective against a Broad Spectrum of Highly Antibiotic-Resistant Superbugs. ACS Chem. Biol..

[21] Torrent M., Andreu D., Nogues V.M., Boix E. (2011). Connecting Peptide Physicochemical and Antimicrobial Properties by a Rational Prediction Model. PLoS ONE.

[22] Mooney C., Haslam N.J., Holton T.A., Pollastri G., Shields D.C. (2013). Peptidelocator: Prediction of Bioactive Peptides in Protein Sequences. Bioinformatics.

[23] Porto W.F., Pires A.S., Franco O.L. (2012). Cs-Amppred: An Updated SVM Model for Antimicrobial Activity Prediction in Cysteine-Stabilized Peptides. PLoS ONE.

[24] Ng X.Y., Rosdi B.A., Shahrudin S. (2015). Prediction of Antimicrobial Peptides Based on Sequence Alignment and Support Vector Machine-Pairwise Algorithm Utilizing LZ-Complexity. Biomed. Res. Int..

[25] Khosravian M., Faramarzi F.K., Beigi M.M., Behbahani M., Mohabatkar H. (2013). Predicting Antibacterial Peptides by the Concept of Chou’s Pseudo-Amino Acid Composition and Machine Learning Methods. Protein Pept. Lett..

[26] Meher P.K., Sahu T.K., Saini V., Rao A.R. (2017). Predicting Antimicrobial Peptides with Improved Accuracy by Incorporating the Compositional, Physico-Chemical and Structural Features into Chou’s General PseAAC. Sci. Rep..

[27] Lira F., Perez P.S., Baranauskas J.A., Nozawa S.R. (2013). Prediction of Antimicrobial Activity of Synthetic Peptides by a Decision Tree Model. Appl. Environ. Microbiol..

[28] Khamis A.M., Essack M., Gao X., Bajic V.B. (2015). Distinct Profiling of Antimicrobial Peptide Families. Bioinformatics.

[29] Xiao X., Wang P., Lin W.Z., Jia J.H., Chou K.C. (2013). Iamp-2l: A Two-Level Multi-Label Classifier for Identifying Antimicrobial Peptides and Their Functional Types. Anal. Biochem..

[30] Maccari G., Di Luca M., Nifosí R., Cardarelli F., Signore G., Boccardi C., Bifone A. (2013). Antimicrobial Peptides Design by Evolutionary Multiobjective Optimization. PLoS Comput. Biol..

[31] Bhadra P., Yan J., Li J., Fong S., Siu S.W.I. (2018). AmPEP: Sequence-based Prediction of Antimicrobial Peptides using Distribution Patterns of Amino Acid Properties and Random Forest. Sci. Rep..

[32] Youmans M., Spainhour C., Qiu P. Long Short-Term Memory Recurrent Neural Networks for Antibacterial Peptide Identification. Proceedings of the 2017 IEEE International Conference on Bioinformatics and Biomedicine (BIBM).

[33] Juretic D., Vukicevic D., Ilic N., Antcheva N., Tossi A. (2009). Computational Design of Highly Selective Antimicrobial Peptides. J. Chem. Inf. Model..

[34] Wang P., Hu L., Liu G., Jiang N., Chen X., Xu J., Zheng W., Li L., Tan M., Chen Z. (2011). Prediction of Antimicrobial Peptides Based on Sequence Alignment and Feature Selection Methods. PLoS ONE.

[35] Melo M.N., Ferre R., Feliu L., Bardaji E., Planas M., Castanho M.A. (2011). Prediction of Antibacterial Activity from Physicochemical Properties of Antimicrobial Peptides. PLoS ONE.

[36] Freire J.M., Dias A.S., Flores L., Veiga A.S., Castanho M.A. (2015). Mining Viral Proteins for Antimicrobial and Cell-Penetrating Drug Delivery Peptides. Bioinformatics.

[37] Chang K.Y., Lin T.P., Shih L.Y., Wang C.K. (2015). Analysis and Prediction of the Critical Regions of Antimicrobial Peptides Based on Conditional Random Fields. PLoS ONE.

[38] Toropova M.A., Veselinovic A.M., Veselinovic J.B., Stojanovic D.B., Toropov A.A. (2015). QSAR Modeling of the Antimicrobial Activity of Peptides as a Mathematical Function of a Sequence of Amino Acids. Comput. Biol. Chem..

[39] Toropov A.A., Toropova A.P., Raska I., Benfenati E., Gini G. (2012). QSAR Modeling of Endpoints for Peptides Which Is Based on Representation of the Molecular Structure by a Sequence of Amino Acids. Struct. Chem..

[40] Lata S., Sharma B.K., Raghava G.P. (2007). Analysis and Prediction of Antibacterial Peptides. BMC Bioinf..

[41] Lata S., Mishra N.K., Raghava G.P. (2010). AntiBP2: Improved Version of Antibacterial Peptide Prediction. BMC Bioinf..

[42] Nagarajan D., Nagarajan T., Roy N., Kulkarni O., Ravichandran S., Mishra M., Chakravortty D., Chandra N. (2018). Computational antimicrobial peptide design and evaluation against multidrug-resistant clinical isolates of bacteria. J. Biol. Chem..

[43] Hincapié O., Giraldo P., Orduz S. (2018). In silico design of polycationic antimicrobial peptides active against Pseudomonas aeruginosa and Staphylococcus aureus. Antonie Van Leeuwenhoek.

[44] Vishnepolsky B., Gabrielian A., Rosenthal A., Hurt D.E., Tartakovsky M., Managadze G., Grigolava M., Makhatadze G.I., Pirtskhalava M. (2018). Predictive model of linear AMPs active against Gram-negative bacteria. J. Chem. Inf. Model..

[45] Vishnepolsky B., Pirtskhalava M. (2019). Comment on: ‘Empirical Comparison of Web-Based Antimicrobial Peptide Prediction Tools’. Bioinformatics.

[46] Vishnepolsky B., Pirtskhalava M. (2014). Prediction of Linear Cationic Antimicrobial Peptides Based on Characteristics Responsible for Their Interaction with the Membranes. J. Chem. Inf. Model..

[47] Nicolau D.P. (2008). Carbapenems: A potent class of antibiotics. Expert Opin. Pharm..

[48] Holton T.A., Pollastri G., Shields D.C., Mooney C. (2013). CPPpred: Prediction of cell penetrating peptides. Bioinformatics.

[49] Matsuzaki K. (1999). Why and how are peptide–lipid interactions utilized for self-defense? Magainins and tachyplesins as archetypes. Biochim. Biophys. Acta (BBA) Biomembr..

[50] Shai Y. (1999). Mechanism of the binding, insertion and destabilization of phospholipid bilayer membranes by α-helical antimicrobial and cell non-selective membrane-lytic peptides. Biochim. Biophys. Acta (BBA) Biomembr..

[51] Yang L., Weiss T.M., Lehrer R., Huang H.W. (2000). Crystallization of antimicrobial pores in membranes: Mgainin and ptegrin. Biophys. J..

[52] Brogden K.A. (2005). Antimicrobial peptides: Pore formers or metabolic inhibitors in bacteria?. Nat. Rev. Microbiol..

[53] Jenssen H., Hamill P., Hancock R.E.W. (2006). Peptide antimicrobial agents. Clin. Microbiol. Rev..

[54] Reddy K.V.R., Yedery R.D., Aranha C. (2004). Antimicrobial peptides: Premises and promises. Int. J. Antimicrob. Agents.

[55] Otvos L. (2000). Antibacterial peptides isolated from insects. J. Pept. Sci..

[56] Brötz H., Bierbaum G., Leopold K., Reynolds P.E., Sahl H.-G. (1998). The lantibiotic mersacidin inhibits peptidoglycan synthesis by targeting lipid II. Antimicrob. Agents Chemother..

[57] Matsuzaki K., Sugishita K.-I., Ishibe N., Ueha M., Nakata S., Miyajima K., Epand R.M. (1998). Relationship of membrane curvature to the formation of pores by magainin 2. Biochemistry.

[58] Dagan A., Efron L., Gaidukov L., Mor A., Ginsburg H. (2002). In vitro antiplasmodium effects of dermaseptin S4 derivatives. Antimicrob. Agents Chemother..

[59] Matsuzaki K., Murase O., Fujii N., Miyajima K. (1996). An antimicrobial peptide, magainin 2, induced rapid flip-flop of phospholipids coupled with pore formation and peptide translocation. Biochemistry.

[60] Wu M., Maier E., Benz R., Hancock R.E.W. (1999). Mechanism of interaction of different classes of cationic antimicrobial peptides with planar bilayers and with the cytoplasmic membrane of *Escherichia coli*. Biochemistry.

[61] Lázár V., Martins A., Spohn R., Daruka L., Grézal G., Fekete G., Számel M., Jangir P.K., Kintses B., Csörgő B. (2018). Antibiotic-resistant bacteria show widespread collateral sensitivity to antimicrobial peptides. Nat. Microbiol..

[62] Shagaghi N., Bhave M., Palombo E.A., Clayton A.H.A. (2017). Revealing the sequence of interactions of PuroA peptide with Candida albicans cells by live-cell imaging. Sci. Rep..

[63] Chan D.I., Prenner E.J., Vogel H.J. (2006). Tryptophan- and arginine-rich antimicrobial peptides: Structures and mechanisms of action. Biochim. Biophys. Acta (BBA) Biomembr..

[64] Patrzykat A., Friedrich C.L., Zhang L., Mendoza V., Hancock R.E.W. (2002). Sublethal concentrations of pleurocidin-derived antimicrobial peptides inhibit macromolecular synthesis in Escherichia coli. Antimicrob. Agents Chemother..

[65] Ester M., Kriegel H., Sander J., Xu X. (1996). A density-based algorithm for discovering clusters in large spatial databases with noise. Proceedings of the Second International Conference on Knowledge Discovery and Data Mining (KDD-96).

[66] Moon C.P., Fleming K.G. (2011). Side-chain hydrophobicity scale derived from transmembrane protein folding into lipid bilayers. Proc. Natl. Acad. Sci. USA.

[67] Senes A., Chadi D.C., Law P.B., Walters R.F.S., Nanda V., DeGrado W.F. (2007). E(z), a Depth-dependent Potential for Assessing the Energies of Insertion of Amino Acid Side-chains into Membranes: Derivation and Applications to Determining the Orientation of Transmembrane and Interfacial Helices. J. Mol. Biol..

[68] Uversky V., Gillespie J., Fink A. (2000). Why are “natively unfolded” proteins unstructured under physiological conditions?. Proteins Struct. Funct. Gen..

[69] Kessel A., Ben-Tal N. (2011). Introduction to Proteins: Structure, Function and Motion (Chapman & Hall/CRC Mathematical and Computational Biology).

[70] The UniProt Consortium (2015). UniProt: A hub for protein information. Nucleic Acids Res..

[71] Wang G., Li X., Wang Z. (2016). APD3: The antimicrobial peptide database as a tool for research and education. Nucleic Acids Res..

[72] Waghu F.H., Barai R.S., Gurung P., Idicula-Thomas S. (2016). CAMPR3: A database on sequences, structures and signatures of antimicrobial peptides. Nucleic Acids Res..

[73] Fan L., Sun J., Zhou M., Zhou J., Lao X., Zheng H., Xu H. (2016). DRAMP: A comprehensive data repository of antimicrobial peptides. Sci. Rep..

[74] Wiegand I., Hilpert K., Hancock R.E.W. (2008). Agar and broth dilution methods to determine the minimal inhibitory concentration (MIC) of antimicrobial substances. Nat. Protocol..

