# Supplementary material for: De Novo Design and In Vitro Testing of Antimicrobial Peptides against Gram-Negative Bacteria
Source: Pharmaceuticals (Basel). 2019 Jun 3;12(2):82. doi: 10.3390/ph12020082 (PMC6631481; doi:10.3390/ph12020082)
Supplement: Supplementary file 1 [file pharmaceuticals-12-00082-s001.pdf]

Supplementary Materials

De-novo design and in vitro testing of the antimicrobial peptides against Gram-negative bacteria

Boris Vishnepolsky [1,*], George Zaalishvili [2], Margarita Karapetian [2], Tornike Nasrashvili [2], Nato Kuljanishvili [2], Andrei Gabrielian [3], Alex Rosenthal [3], Darrell E. Hurt [3], Michael Tartakovsky [3], Maya Grigolava [1], Malak Pirtskhalava [1,*]

[1] Ivane Beritashvili Center of Experimental Biomedicine, Tbilisi 0160, Georgia; b.vishnepolsky@lifescience.org.ge (B.V.); maia.grigolava@science.org.ge (M.G.); m.pirtskhalava@lifescience.org.ge (M.P.)

[2] Agricultural University of Georgia, 240 David Aghmashenebeli Alley, Tbilisi 0159, Georgia gi.zaalishvili@agruni.edu.ge (G.Z.); m.karapetian@agruni.edu.ge (M.K.); tnasr2015@agruni.edu.ge (T.N.); nkulj2014@agruni.edu.ge (N.K.)

[3] Office of Cyber Infrastructure and Computational Biology, National Institute of Allergy and Infectious Diseases, National Institutes of Health, Bethesda, MD 20892, USA; gabr@niaid.nih.gov (A.G.); alexr@niaid.nih.gov (A.R.); darrellh@niaid.nih.gov (D.E.H.); mtartakovs@niaid.nih.gov (M.T.)

* Correspondence: b.vishnepolsky@lifescience.org.ge (B.V); m.pirtskhalava@lifescience.org.ge (M.P.)

Content

1. Screenshot of DBAASP Ranking Search Page (**Figure S1)**
2. Amino acid distribution used in the sequence generation algorithm (**Table S1)**

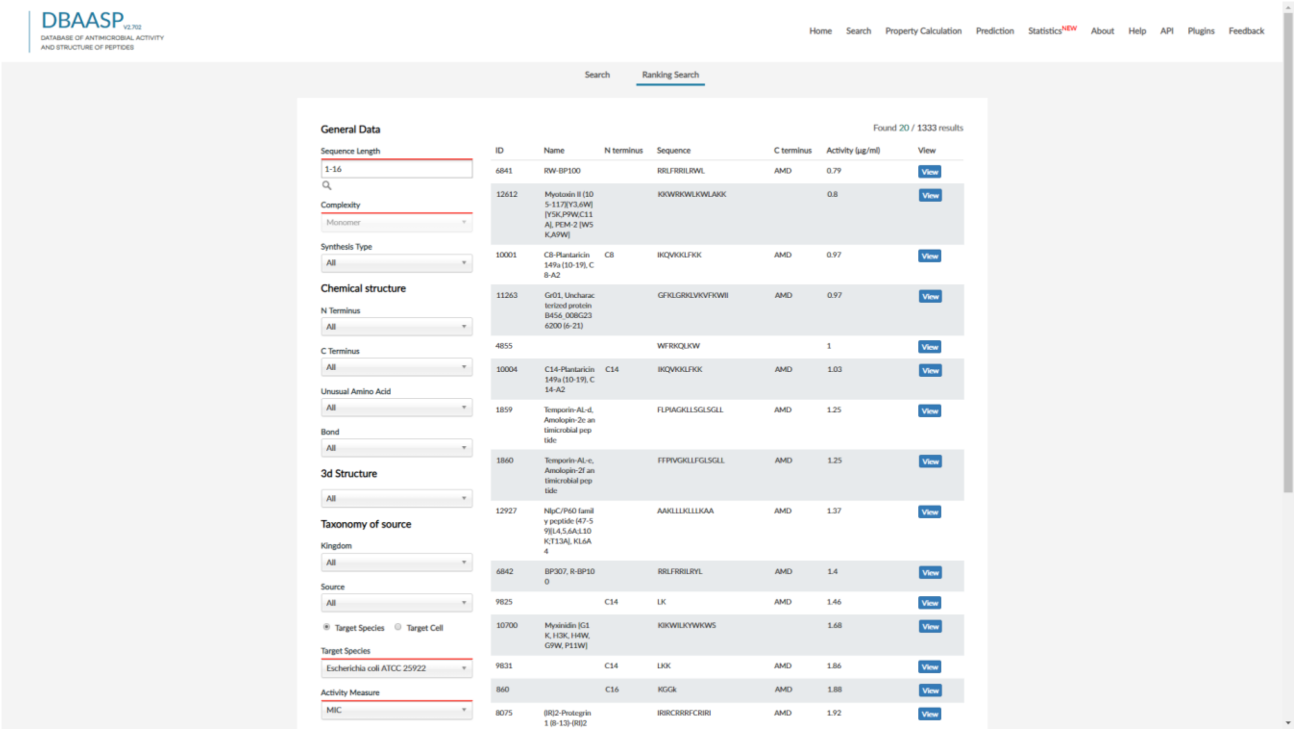

Figure S1. Screenshot of DBAASP Ranking Search Page for peptides with length <17 for which there are data for activity against *Escherichia coli ATCC 25922*.

As we can see, the first ranking peptide has MIC=0.79. At the same time for peptide SP15D MIC=0.39-0.78.

Table S1. Amino acid distribution for the set of linear, ribosomal AMPs from the data of DBAASP.

Amino Acid	Frequency
C	0.909
N	3.169
Q	2.484
S	5.040
T	2.766
D	2.198
E	2.593
K	11.607
R	5.403
A	9.333
G	12.449
I	7.624
L	11.232
M	1.295
P	4.855
V	6.091
F	4.889
H	2.503
W	1.734
Y	1.633
